# An Innovative AI-based primer design tool for precise and accurate detection of SARS-CoV-2 variants of concern

**DOI:** 10.1038/s41598-023-42348-y

**Published:** 2023-09-22

**Authors:** Carmina Angelica Perez-Romero, Lucero Mendoza-Maldonado, Alberto Tonda, Etienne Coz, Patrick Tabeling, Jessica Vanhomwegen, John MacSharry, Joanna Szafran, Lucina Bobadilla-Morales, Alfredo Corona-Rivera, Eric Claassen, Johan Garssen, Aletta D. Kraneveld, Alejandro Lopez-Rincon

**Affiliations:** 1 Departamento de Investigación, Universidad Central de Queretaro (UNICEQ), Av. 5 de Febrero 1602, San Pablo, Santiago de Querétaro, 76130 Qro. Mexico; 2 Hospital Civil de Guadalajara “Dr. Juan I. Menchaca”, Salvador Quevedo y Zubieta 750, Independencia Oriente, C.P. 44340 Guadalajara, Jalisco México; 3 UMR 518 MIA Paris-Saclay, INRAE, AgroParisTech, Université Paris-Saclay, 91120 Palaiseau, France; 4 CBI, ESPCI Paris, Université PSL, CNRS, 75005 Paris, France; 5 CIBU, Institut Pasteur, 25-28 rue du Dr Roux, 75015 Paris, France; 6 School of Microbiology and School of Medicine, University College Cork, College Rd, University College, Cork, Ireland; 7 Athena Institute, Vrije Universiteit, De Boelelaan 1085, 1081 HV Amsterdam, The Netherlands; 8 Division of Pharmacology, Utrecht Institute for Pharmaceutical Sciences, Faculty of Science, Utrecht University, Universiteitsweg 99, 3584 CG Utrecht, The Netherlands; 9 Department Immunology, Danone Nutricia research, Uppsalalaan 12, 3584 CT Utrecht, The Netherlands; 10 Julius Center for Health Sciences and Primary Care, University Medical Center Utrecht, Universiteitsweg 100, 3584 CG Utrecht, The Netherlands

**Keywords:** Classification and taxonomy, Biomedical engineering, Machine learning

## Abstract

As the COVID-19 pandemic winds down, it leaves behind the serious concern that future, even more disruptive pandemics may eventually surface. One of the crucial steps in handling the SARS-CoV-2 pandemic was being able to detect the presence of the virus in an accurate and timely manner, to then develop policies counteracting the spread. Nevertheless, as the pandemic evolved, new variants with potentially dangerous mutations appeared. Faced by these developments, it becomes clear that there is a need for fast and reliable techniques to create highly specific molecular tests, able to uniquely identify VOCs. Using an automated pipeline built around evolutionary algorithms, we designed primer sets for SARS-CoV-2 (main lineage) and for VOC, B.1.1.7 (Alpha) and B.1.1.529 (Omicron). Starting from sequences openly available in the GISAID repository, our pipeline was able to deliver the primer sets for the main lineage and each variant in a matter of hours. Preliminary in-silico validation showed that the sequences in the primer sets featured high accuracy. A pilot test in a laboratory setting confirmed the results: the developed primers were favorably compared against existing commercial versions for the main lineage, and the specific versions for the VOCs B.1.1.7 and B.1.1.529 were clinically tested successfully.

## Introduction

Severe acute respiratory syndrome coronavirus 2 (SARS-CoV-2) was identified in December 2019 in Wuhan, China^1^, as a new strain of coronavirus that causes COVID-19 disease^2^. Since then, COVID-19 has evolved into a pandemic with nearly 540 million confirmed cases and over 6.3 million deaths worldwide^3^, as of June 2022. Although now vaccines are available for SARS-CoV-2, the outbreak still represents a massive challenge because of the characteristics of the COVID-19 disease, e.g. long incubation period, wide range of symptoms, high infection rate, high false negative rate (FNR) in detection tests and high mutation rate^4^.

An early estimate of SARS-CoV-2 mutation rate is $1.12\times 10^{-3}$ mutations per site-year^5^. The high mutation rate of SARS-CoV-2, resulted in different variants, where some of them showed a higher rate of transmissibility, virulence, clinical presentation, mortality and/or vaccine/therapeutics resistance^6^. Given their acquired mutations, and aforementioned characteristics some variants were deemed variants of concern (VOCs) by the WHO. The previously circulating VOCs were B.1.1.7 (Alpha), B.1.351 (Beta), P.1 (Gamma) and B.1.617.2 (Delta) in Pango lineage^7^. These were designated as previous VOCs on 09-Mar-2022 (B.1.1.7, B.1.351 and P.1) and 7-Jun-2022 (B.1.617.2). At present the dominant VOC circulating in the world stems from B.1.1.529 (Omicron) designated on 26-Nov-2021, including the sublineages BA.1, BA.2, BA.3, BA.4 and BA.5^8^. The initial Omicron outbreak was caused by BA.1. More than 60 non-synonymous mutations were discovered in the BA.1 and BA.2 variants, according to a whole-genome sequencing analysis, including base substitutions, insertions, and deletions.

During a pandemic, having access to a precise diagnosis tool can help decision-makers take appropriate measures, e.g. isolation, monitoring and quarantine of patients to reduce infections^9^. Nevertheless, given the magnitude of the outbreak, health systems and health workers found themselves overwhelmed by the number of patients needed to be tested. As a possible solution, several research lines proposed the use of artificial intelligence (AI) to automate the detection of infections^10^. Despite the great potential of AI, a recent meta-analysis of these methodologies strikingly found that classical methods, such as Quantitative reverse transcription PCR (RT-qPCR), have been more accurate during the COVID-19 pandemic, than AI-based detection techniques, e.g. diagnosis based on imaging^11^.

RT-qPCR is both an effective tool to identify SARS-CoV-2 infections, and the most widespread approach to diagnose COVID-19 in symptomatic and asymptomatic patients^12^. In summary, the methodology requires a sample from the patient, and then a specific sequence of the viral genome is targeted. If the sample contains the expected sequence, then this will start the polymerase chain reaction to amplify the virus-specific sequence and thereby the virus will be detected. Nevertheless, the high mutation rate of SARS-CoV-2, made it difficult to design tests for each of the VOCs. For example, VOC B.1.1.7 was identified through an increase in the S-gene target failure in a three-target gene assay (N+, ORF1ab+, S−), coupled with sequencing of the virus and RT-qPCR amplicons products^13^. The S-gene target failure occurs when one of the RT-qPCR probes fails to bind, as a result of the $\bigtriangleup$69−70 deletion in the SARS-CoV-2 spike protein, present in B.1.1.7^13^. This $\bigtriangleup$69−70 deletion, which affects its N-terminal domain, has been occurring in several different SARS-CoV-2 variants around the world^14,15^ and has been associated with other spike protein receptor binding domain changes^16^. This is consistent with other existing primer designs like CoV2R-3 in the S-gene^17^, that will also yield negative results for the B.1.1.7 variant, as the reverse primer sequence is in the region of mutation P681H. A more in-depth analysis of S-dropout positive results can be found in Kidd et al.^18^. Due to the likeliness of mutations in the S-gene, assays relying solely on its detection are no longer recommended, and a multiplex approach is required to detect SARS-CoV-2 and the VOCs^19–24^.

As the pandemic evolved and new VOCs appeared, it became clear that we need more specific and efficient primer design to detect the virus and to compensate for the high mutation rate were necessary. We propose an approach based on evolutionary algorithms (EAs)^25^, an AI technique, and available sequences from the Global Initiative on Sharing Avian Influenza Data (GISAID)^26^, to find specific sub-sequences that could be used as primers for RT-qPCR to detect SARS-CoV-2 and VOCs in human samples. The basic idea of the approach is to uncover 21-bps sequences specific to a given virus strain (in this case SARS-CoV-2 or a variant), rank them by their suitability as primers, and measure their capacity of discriminating samples belonging to the target virus from other strains. In a previous work, we generated a set of primers to detect SARS-CoV-2 with Convolutional Neural Networks (CNNs)^25^ using 52,645 SARS-CoV-2 sequences from the GISAID database and 20,572 sequences of other taxa from the National Center for Biotechnology Information (NCBI) database^27^. Then, we tested the resulting sequences in silico using the software Primer3Plus^28^ to verify their suitability as primer sets using the SARS-CoV-2 reference genome NC045512 from NCBI^29^. The process generated a primer set in the *ORF3a* gene of the SARS-CoV-2 genome, that was named *UtrechtU-ORF3a*, and was successfully tested in clinical settings with 10 patients’ samples^25^. Then, we improved the AI method to be faster, and at the same time specific enough to generate primer sets for the VOCs^30^.

In the context of justified skepticism of AI-based techniques, we then decided to perform a more robust validation of the RTq-PCR diagnostic tool we developed using AI. First, we clinically validated our main lineage primer *UtrechtU-ORF3a* by comparing them against 2 commercial kits for 20 patients’ samples, 15 positive and 5 negative. Next, we designed specific primer sets for VOCs B.1.1.7 (Alpha) and B.1.1.529 (Omicron) and clinically validated them against government approved tests. The proof-of-concept of our AI-Based Primer Design Tool can aid in the creation of accurate detection RT-qPCR tools for this and future pandemics.

## Results

### Main lineage comparison to commercial kits

Nasopharingeal and oropharingeal samples were evaluated with three different PCR tests: the *UtrechtU-ORF3a*, *DeCoV19 Kit Triplex* and *GeneFinder COVID-19 PLUS RealAmp Kit* primers. The *UtrechtU-ORF3a* primer set amplified correctly 15 positive samples with cycle threshold (CT) values similar to *DeCoV19 Kit Triplex* and *GeneFinder COVID-19 PLUS RealAmp Kit* primers (Table 1). Conversely, the 5 negative samples were correctly undetected by all three primer kits. We found that our primer set had a comparable performance and demonstrated its specificity and accuracy in detecting SARS-CoV-2 infections.

**Table 1:** CT (cylce threshold) values obtained from AI primers (Utrecht primers) and commercial kits (Genefinder, Decov-19), where positive SARS-CoV-2 samples are marked as $(+)$, and negative samples are marked as $(-)$. E, RdRP, N, IC are the targets of Genefinder. N1, N2, N3 are the targets of Decov-19. 1, 2 are the replicates of the UtrechtU-ORF3a primers.

Sample	Genefinder		Decov-19		Utrecht primers		Sample	Genefinder		Decov-19		Utrecht primers	
1	E:	17.5	N1:	18.3	1:	17.2	11	E:	40	N1:	34.2	1:	40
(+)	RdRp:	20.6	N2:	19.2	2:	17.3	(+)	RdRp:	40	N2:	34.8	2:	36.7
	N:	17.5	N3:	16.8				N:	40	N3:	33.2		
	IC:	23.3	RnaseP:	25				IC:	27.8	RnaseP:	32.2		
2	E:	16	N1:	14.7	1:	15.4	12	E:	27.2	N1:	24.9	1:	27.4
(+)	RdRp:	18.9	N2:	16.9	2:	15.2	(+)	RdRp:	29.9	N2:	25.9	2:	27.5
	N:	17.3	N3:	14.4				N:	28.5	N3:	24.5		
	IC:	21.5	RnaseP:	22				IC:	28.8	RnaseP:	28.5		
3	E:	40	N1:	34.7	1:	36.7	13	E:	21.2	N1:	21.1	1:	20.7
(+)	RdRp:	40	N2:	37.2	2:	36.1	(+)	RdRp:	24.9	N2:	22.2	2:	20.8
	N:	38.9	N3:	34				N:	22.4	N3:	20.1		
	IC:	26.7	RnaseP:	25.6				IC:	25.3	RnaseP:	25.9		
4	E:	25.5	N1:	25.9	1:	25.4	14	E:	22.6	N1:	22.2	1:	22.5
(+)	RdRp:	28.9	N2:	27.4	2:	25.6	(+)	RdRp:	25.6	N2:	23.5	2:	22
	N:	26.1	N3:	25				N:	23	N3:	21.9		
	IC:	26	RnaseP:	25.1				IC:	26.8	RnaseP:	28.4		
5	E:	24	N1:	20.3	1:	23.9	15	E:	–	N1:	–	1:	–
(+)	RdRp:	27.2	N2:	23.3	2:	24	(−)	RdRp:	–	N2:	–	2:	–
	N:	29	N3:	20.3				N:	–	N3:	–		
	IC:	23.5	RnaseP:	28.4				IC:	26.6	RnaseP:	25.3		
6	E:	–	N1:	–	1:	–	16	E:	28.7	N1:	27.4	1:	28.8
(−)	RdRp:	–	N2:	–	2:	–	(+)	RdRp:	31.6	N2:	28.6	2:	28.6
	N:	–	N3:	–				N:	29.1	N3:	26.8		
	IC:	24.7	RnaseP:	22.9				IC:	26.8	RnaseP:	25.1		
7	E:	36	N1:	35.6	1:	34.3	17	E:	25.8	N1:	25.1	1:	25.6
(+)	RdRp:	40	N2:	37.3	2:	34.7	(+)	RdRp:	28.4	N2:	25.9	2:	25.5
	N:	34.9	N3:	35.5				N:	26.3	N3:	24.3		
	IC:	27.7	RnaseP:	28.2				IC:	25.2	RnaseP:	25.7		
8	E:	–	N1:	–	1:	–	18	E:	40	N1:	34.2	1:	35.2
(−)	RdRp:	–	N2:	–	2:	–	(+)	RdRp:	40	N2:	34.9	2:	35.3
	N:	–	N3:	–				N:	33.7	N3:	32.8		
	IC:	26.9	RnaseP:	25.2				IC:	25.3	RnaseP:	24.7		
9	E:	–	N1:	–	1:	–	19	E:	35	N1:	31.9	1:	32.4
(−)	RdRp:	–	N2:	–	2:	–	(+)	RdRp:	40	N2:	32.6	2:	32.6
	N:	–	N3:	–				N:	35	N3:	30.1		
	IC:	26	RnaseP:	25.4				IC:	20.1	RnaseP:	24.2		
10	E:	25	N1:	23.7	1:	24.4	20	E:	–	N1:	–	1:	–
(+)	RdRp:	28.1	N2:	24.5	2:	24.3	(−)	RdRp:	–	N2:	–	2:	–
	N:	25.8	N3:	22.9				N:	–	N3:	–		
	IC:	25.7	RnaseP:	25.6				IC:	25.3	RnaseP:	28.8		

**Figure 1:**
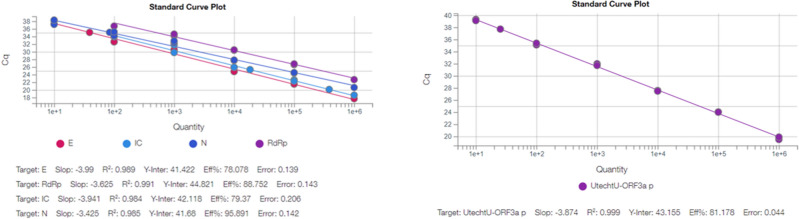
*GeneFinder COVID-19 Plus RealAmp* standard viral load curve (Left). *UtrechtU-ORF3a* standard viral load curve (Right).

Next, we compared viral loads from the 15 positive samples using *GeneFinder COVID-19 PLUS RealAmp Kit* and *UtrechtU-ORF3a* primer set (Fig. 1). For *GeneFinder COVID-19 PLUS RealAmp Kit*, we used *E* gene CT values for comparison. We observed that there are variations in log and copies/µl between methodologies. For the total of positive samples, the viral load measurement was higher using the *UtrechtU-ORF3a* primers. On the other hand, when interpolating the E gene CT values in the standard curve, it was observed that the result was less than 1 copy/µl in samples 3, 11, and 18 using the *GeneFinder COVID-19 PLUS RealAmp Kit*. This shows that UtrechtU-ORF3a primer set had a comparable or superior sensitivity compared to Genefinder, where lower viral loads are the limiting factor for accurate detection of SARS-CoV-2 infection, as seen in patient sample 11.

### Experimental evaluation of B.1.1.7 primer specificity

**Figure 2:**
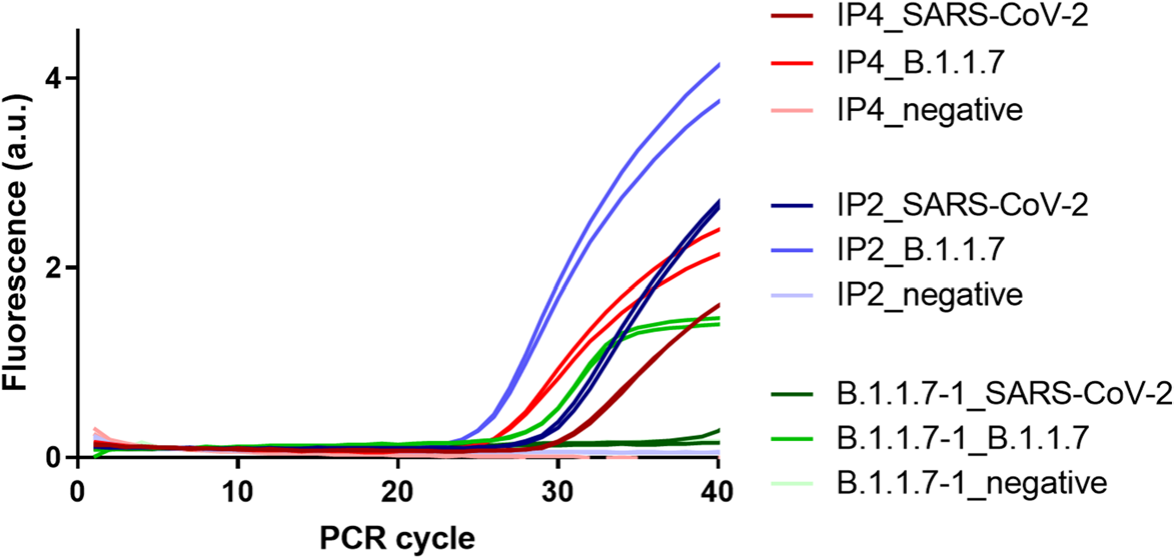
Comparison between non-specific primer set IP2 and IP4 and our designed primer set B.1.1.7-1 for B.1.1.7 variant and others.

The RT-qPCR amplification curves obtained with B.1.1.7 specific primer set *B.1.1.7-1* generated using AI and generic SARS-CoV-2 primers IP2 (RdRp gene/nCoV_IP2) and IP4 (RdRp gene / nCoV_IP4), following the Pasteur Institute Protocol^31^ on two SARS-CoV-2 strains are shown in Fig. 2. As expected, only the B.1.1.7 strain is amplified by the B.1.1.7-1 primers, while both the Wuhan reference strain (NC_045512.2) and the B.1.1.7 strain are detected by the generic primers (Table 2).

**Table 2:** Cycle threshold (CT) values obtained from the specific B.1.1.7 (Alpha) primer set *B.1.1.7-1*, where positive B.1.1.7 samples are marked as $(+)$, and negative control and other lineage samples are marked as $(-)$.

Sample	B.1.1.7-1	IP2	IP4
(-) SARS-CoV-2	–	25	31.44
(+) B.1.1.7	28.44	29.56	26.94
(-) Negative	–	–	–

### Experimental evaluation of B.1.1.529 primer specificity

Laboratory testing of the generated specific primer set *B.1.1.529-1*, as part of the UniCoV study^32^, where we used raw saliva samples, proved to be successful (see Table 3) in identifying the Omicron variant. Thus, these results further demonstrated that the use of AI to generate specific diagnostic tests can be used as a fast measure to tackle the on-going SARS-CoV-2 pandemic and the appearance of new VOCs.

**Table 3:** Cycle threshold (CT) values obtained from the generated B.1.1.529 (Omicron) specific primers *B.1.1.529-1*, where positive SARS-CoV-2 samples are marked as $(+)$, and negative samples are marked as $(-)$. N1 is the 2019-nCoV-N1 primer set. The sample names remain the same as in the Unicov study^32^.

Sample	N1	B.1.1.529-1
(+) 1515	33.96	37.00
(+) 4702	28.79	33.04
(-) 100308	36.06	–
(+) 124546	26.61	32.37
(-) 1122021a	29.10	–
(+) 105012022	28.69	36.51
(-) Negative (dH2O)	–	–
(-) RT-Negative	–	–

## Discussion

Since the COVID-19 pandemic was declared in March 2020^33^, new VOCs, such as B.1.1.7(Alpha), B.1.351 (Beta)^34^, P.1 (Gamma)^35^, B.1.617.2 (Delta)^36^ and B.1.1.529 (Omicron)^37^, have been emerging. As the SARS-CoV-2 mutated, some of the first primer sets designs failed to detect the mutated virus and certain characteristics made it necessary to separate the different lineages, this can be seen in supplementary figure 1. Thus, it was necessary to develop new VOC-specific primer sets as fast as possible and specific to certain variants. As a solution, we propose to use AI, and specifically Evolutionary Algorithms (EAs), to develop a tool to find candidate VOC-specific primers.

Our experiments show that the specificity and sensitivity of our AI-based primers can be a viable way of detecting SARS-CoV-2 but also specific VOCs, proving the usefulness of AI techniques in clinical settings. Our methodology is not only applicable to the design of primer sets to detect SARS-CoV-2 and its variants, but can also be used for other upcoming viruses, as long as a minimum of 10 viral sequences are available^38^. Critiques related to the use of AI in medicine often focus on the poor replicability of the results, on the lack of proper follow-ups with laboratory validation, or on the absence of comparison against more traditional techniques^11,39,40^. In order to improve the state of the art in the AI field, we performed a thorough validation of our AI-based technique, in a clinical setting involving 20 patients and a comparison against commercial diagnostic kits (for primers developed for the main SARS-CoV-2 lineage); for variant primers, we tested their specificity in laboratory settings for B.1.1.7 (Alpha) and B.1.1.529 (Omicron). Although, a limitation of our study is that we need to make further analyses with more samples from the VOCs.

We believe that all the proposed primer sets can be employed in a multiplexed approach in samples for the initial diagnosis of COVID-19 patients, or used as a second step of diagnosis in cases already verified positive to SARS-CoV-2, to identify specific VOCs. In addition, the methodology can be in principle applied to other detection techniques, such as loop-mediated isothermal amplification (LAMP), but this will require further testing.

In this way, health authorities can better evaluate the medical outcomes of patients, and adapt or inform new policies that can help curve the rise of variants of interest, and new potential viruses. For example, in May 2022, several cases of a human Monkeypox virus were identified outside endemic countries^41^, and has since been spreading across the globe prompting the WHO to declare it a global emergency last 23 of July 2022^42^. Thus, using our AI-based methodology and 191 Monkeypox sequences available in the GISAID repository, along with 20,603 sequences from other viruses from the NCBI dataset, we obtained 4 different primer sets with 100% in-silico specificity and 100% sensitivity using sequence EPI_ISL_13053218 as reference. The results are reported in supplementary table 5. Although future primer sets delivered by our automated methodology will still require laboratory testing to be validated, our methodology can enable the timely, rapid, and low-cost operations needed for the design of new primer sets to accurately diagnose new emerging SARS-CoV-2 variants and emerging viral infectious diseases.

## Methods

**Figure 3:**
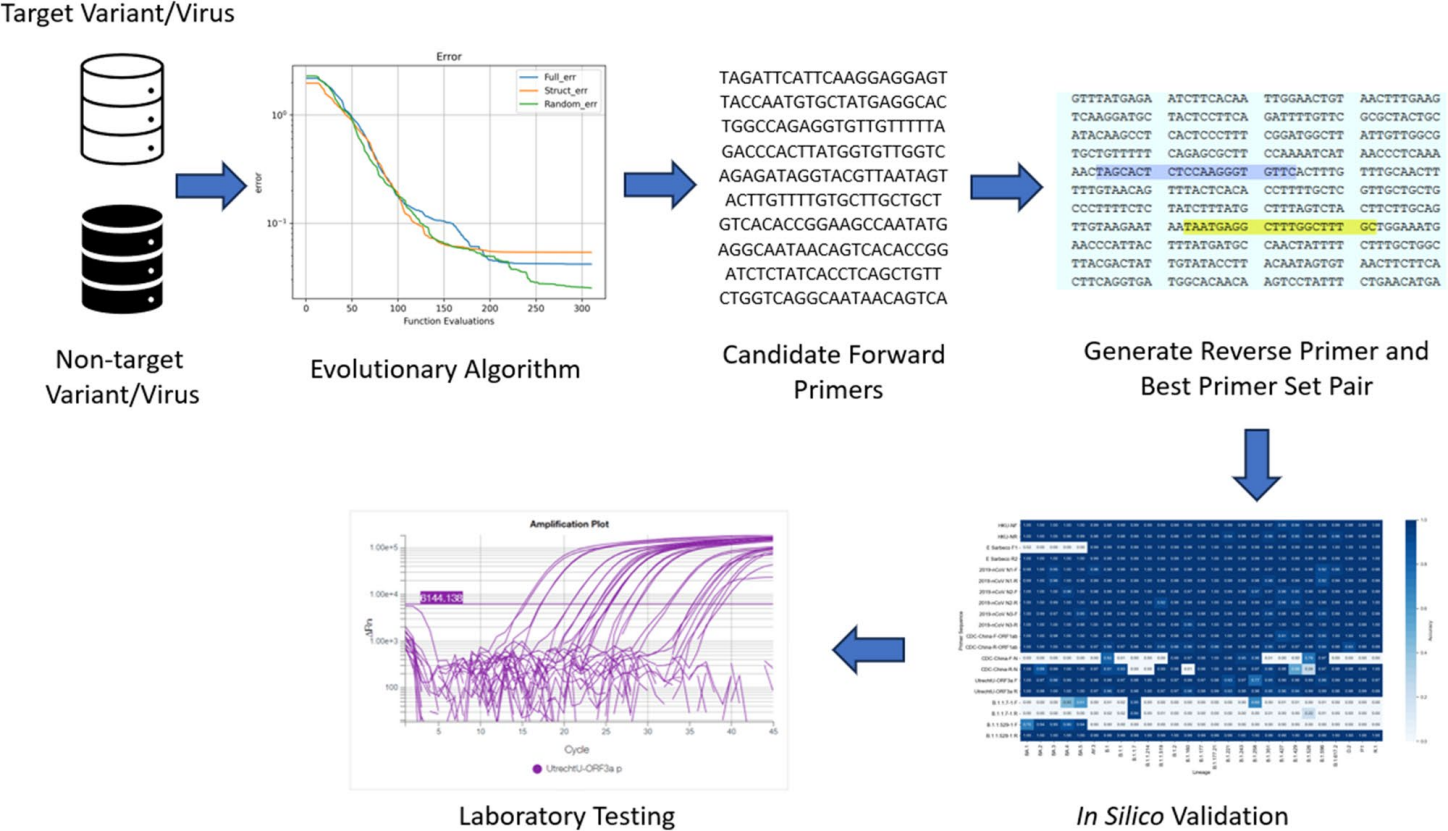
Summary of the procedure to create, validate and test a primer set designed using evolutionary algorithms.

The methodology to create a primer set is summarized in Fig. 3. First, we generate a dataset containing the target viral sequence labeled as “1” and other viral sequences labeled as “0”. In the case of the main lineage we will have a dataset with SARS-CoV-2 sequences labeled as “1” and other viruses labeled as “0”. For the VOCs we create the dataset with the target variant as “1” and a set of different lineages of SARS-CoV-2 lineages as “0”. Then, using evolutionary algorithms we find a candidate forward primer, by finding a suitable 21-bps sequence that can distinguish the target variant/virus from the rest. Next, assisted by Primer3Plus^28^, we verify that the forward primer is viable and create the reverse primer. In the case of SARS-CoV-2, we validate the generated primers in silico in 2,107,300 SARS-CoV-2 sequences from the GISAID repository. Finally, we test the primer set in laboratory settings.

### Evolutionary algorithm

We will present a summary of the use of artificial intelligence to generate the different primer sets to detect the main lineage of SARS-CoV2, and the VOCs B.1.1.7 and B.1.1.529. A more detailed description is available in^25^. Evolutionary Algorithms (EAs) are a stochastic optimization technique, able to find candidate solutions that maximize or minimize a given cost function for a specific problem. EAs are suited for searching among a vast number of different alternatives, that would be impossible to process exhaustively. In our case, candidate solutions are 21-bps sub-sequences found inside samples or sequences of the target virus of lineage, e.g. sub-sequences of B.1.1.7, that separate it from the other lineages. In our particular case, the cost function will be a measure of how probable is that a found 21-bps sequence in a position *p* in a sample *k* could be used as a forward primer. This given measure considers the GC content, no missing values (N) in the sequence, the specificity and the temperature of the found forward primer.

The cost function in our study evaluates the suitability of a candidate sub-sequence as primer, is to be maximized, and is given by the following:

1 $$\begin{aligned} F(I)=w_p \cdot \textit{Specificity}(I) + w_c \cdot \textit{GC}(I) + w_n \cdot \textit{N}_{val}(I) + w_t \cdot \textit{Temp}(I) \end{aligned}$$

with $w_p, w_c, w_n, w_t$ representing the weights associated to each term.

2 $$\begin{aligned} \textit{Specificity}(I)=\sum _{i=0}^{T}{P(I, s_i)}, \end{aligned}$$

$\textit{Specificity}(I)$ is evaluating the presence of the sequence selected as candidate primer *I* inside training samples labeled with the variant of interest, and its absence from samples of other variants, *T* is the number of samples in the training set, $s_i$ is the *i*-th sample in the training set. Function *P* is defined as:

3 $$\begin{aligned} P(I, s_i) = {\left\{ \begin{array}{ll} 0,&{} \text {if }I\text { is found inside }s_i\text { and }L(s_i) == L(s_k) \\ 1,&{} \text {otherwise}. \end{array}\right. } \end{aligned}$$

where *L*(*s*) returns the class label of sample *s*. In other words, $P(I,s_i)$ equals 1 if sequence *I* is found inside a sample with the same class label as sample $s_k$, the origin of sequence *I*. So, if the 21-bps sequence *I* is found inside a sample that does not belong to the variant of interest, or is not found in a sample that belongs to the variant of interest, the solution is penalized.

The second term of the weighted sum takes into account the GC content of the candidate primer:

4 $$\begin{aligned} \textit{GC}(I) = 0.5 - \sum _{i=0}^{21}{\dfrac{C(I(i))}{21}}&\text {where }C(b)= {\left\{ \begin{array}{ll} 1,&{} \text {if base }b\text { is C or G} \\ 0,&{} \text {otherwise}. \end{array}\right. } \end{aligned}$$

where *I*(*i*) represents the base in position *i* inside sequence *I*. $\textit{N}_{val}$ is defined as the following equation, that takes into account the presence of N symbols in the sequence, indicating an error in the read. The ideal primer candidate should only contain A, C, G, or T values.

5 $$\begin{aligned} \textit{N}_{val}(I) = \sum _{i=0}^{21}{N(I(i))}&\text {where}&N(b) = {\left\{ \begin{array}{ll} 1,&{} \text {if base }b\text { is N} \\ 0,&{} \text {otherwise}. \end{array}\right. } \end{aligned}$$

The final term tackles the requirement of having a melting temperature $T_m$ centered around $60^{\circ }$. Specialized literature^28^ provides an equation to compute $T_m$ for a sequence *I*:

6 $$\begin{aligned} T_m(I) = 81.5 + 16.6 * log_{10}([Na+]) + 41 * \textit{GC}(I) - 600 / l(I) \end{aligned}$$

where $\textit{GC}(I)$ is the content of C and G bases in sequence *I*, as described in Equation 4, $[Na+]$ is the molar sodium concentration, and *l*(*I*) is the length of sequence *I*, in bps. We used the value of $[Na+]=0.2$ as described in^28^, while $l(I)=21$ by design. The term taking into account $T_m$ will then be:

7 $$\begin{aligned} \textit{Temp}(I)=|60-T_m(I)| \end{aligned}$$

**Figure 4:**
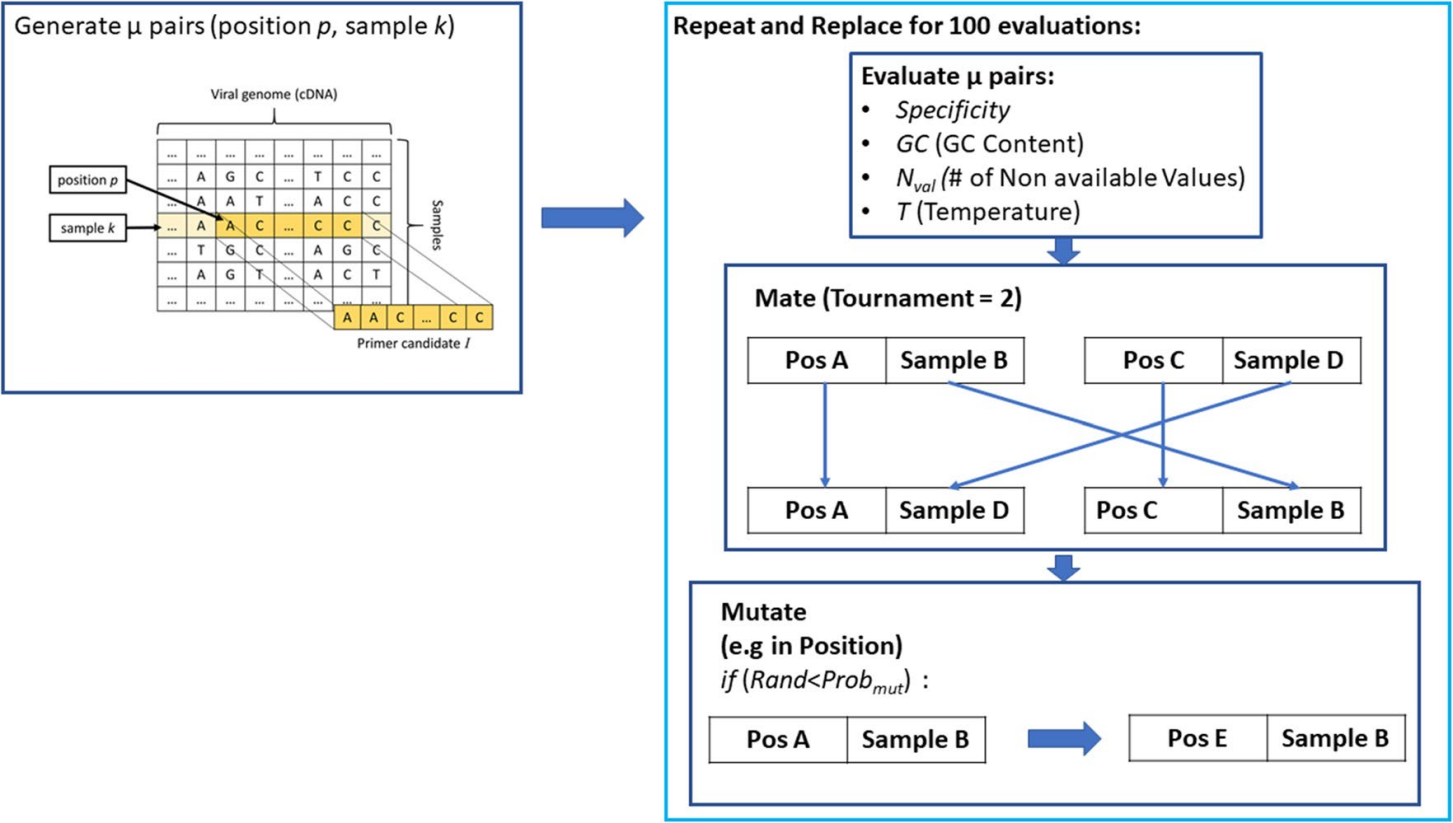
Summary of the EA where we expect to find a target 21-bps sequence in position *p* and in sample *k* to be used as a forward primer.

The EA is set with a population of size $\mu =200$, generating offspring of size $\lambda =200$. The entire population is replaced by its offspring at each generation, using a ($\lambda ,\mu$) replacement strategy, with a tournament selection of size $\tau =2$, a mutation acting on integer values, a one-point crossover, and a stop condition set on 100 generations, the value of 100 was selected from prelimonary testing and comparison to the results in^30^. A summary of the used EA is in Fig. 4. In contrast to previous methods for finding primers, that used Convolutional Neural Networks (CNN)^25^, this approach has the advantage of reducing the time required to obtain candidates. In comparison to the 16 hours required by the CNN approach, each single run of the EA lasts around 62 minutes with 5 threads on a 64-bit Windows 10 laptop with an Intel Xeon E-2186M microprocessor. Furthermore, CNN-based approaches require a considerable amount of post-processing to filter out sub-sequences without the desired requirements as primers, while the cost function already selects for most of the necessary requirements.

### In-silico validation of the designed primers

After we have created the forward primer using the EAs, we will verify it using and generate the reverse primer with Primer3Pus^28^ in the reference sequences for each virus or variant, this is done automatically by our tool. Then, we test for specificity in silico. This test involves verifying if the sequence (or sample) contains the targeted sequences from the primer set. In comparison to other tools like the Primer-Blast tool^43^, we design the primer set using several sequences at the same time, instead of only one as a reference. For the validation, we will expect that the main lineage primer set *UtrechtU-Orf3a* appears in all of the lineages. In contrast, we expect for the *B.1.1.7-1* and *B.1.1.529-1* primer sets to be specific to the target lineages and not the rest.

For the in silico validation, we downloaded 2,107,300 sequences of SARS-CoV-2 from 27 different lineages and sub-lineages from the GISAID repository, in June $11^{th}$, 2022. The number of sequences by variant of SARS-CoV-2 is available in supplementary table 4.

### Main lineage comparison to commercial kits

#### Create primer set

**Figure 5:**
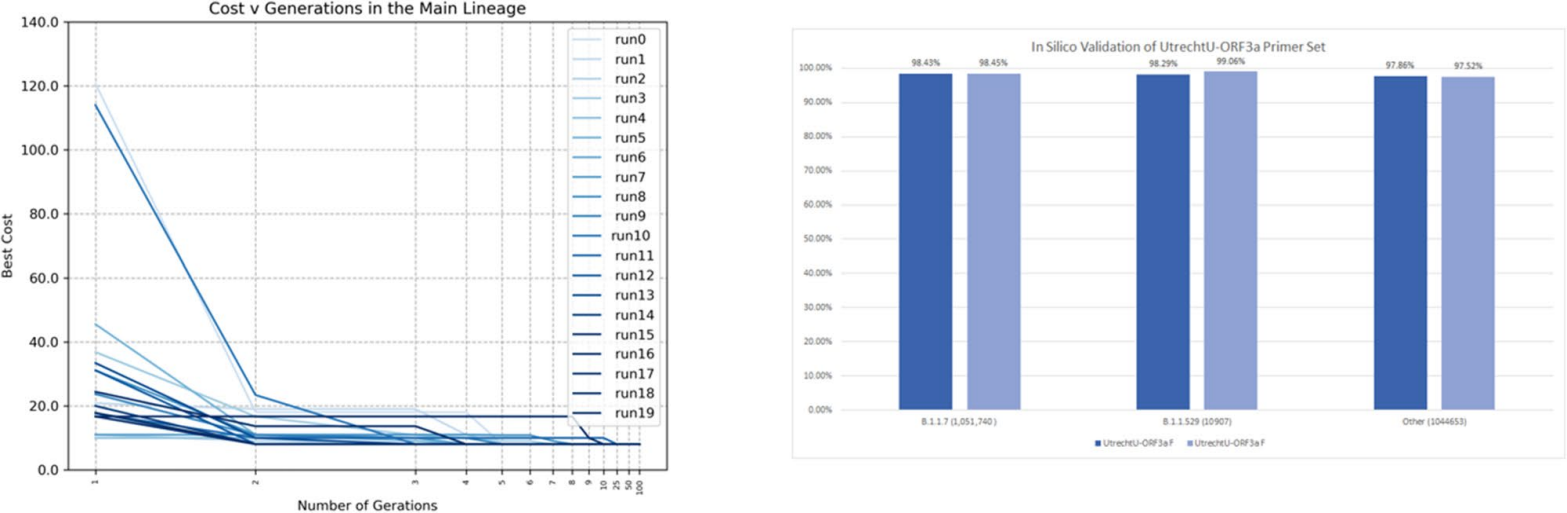
(Left) Cost function in 20 runs of the EA for 100 generations to find a forward primer in the main lineage. (Right) Percentage of appearance of the primer set in the 2,107,300 SARS-CoV-2 sequences.

We downloaded 583 sequences (*.fasta files) from the National Genomics Data Center (NGDC) repository^44^ on March 15th, 2020, with labels: MERS-CoV (236), HCoV-EMC (4), HCoV-OC43 (138), HCoV-229E (22), HCoV-4408 (2), HCoV-NL63 (58), HCoV-HKU1 (17), SARS-CoV (10) and SARS-CoV-2 (96). We assign SARS-CoV-2 as class 1, and the rest as class 0. Then, we run the EA algorithm 20 times, limiting its search to the sequence around gene *ORF3a* from SARS-CoV-2, which yields as candidate forward primers 14 non-repeated sequences. The results of the 20 runs of the EA by generation is in Fig. 5 (left).

The resulting sequences and the Primer3Plus^28^ analysis are in supplementary table 1. From the results, the sequence **5′-TAG CAC TCT CCA AGG GTG TTC-3′** appears 4 times, and can be used as a forward primer and **3′-GCA AAG CCA AAG CCT CAT TA-5′** as reverse primer simulated using Primer3Plus^28^, this is the same result as using a CNN^25^.

#### In silico validation

Although the B.1.1.529 (Omicron) VOC did not exist when we created our primers for the main SARS-CoV-2 lineage (*UtrechtU-ORF3a*), in an initial in-silico analysis of 10,907 Omicron samples from GISAID^26^, the primers return a detection rate above 95% over the 5 Omicron sublineages BA.1-BA.5. In contrast, the E-Sarbeco^45^ (Charite-E) and CHINA-CDC-N primer sets do not detect these Omicron variants, see supplementary figure 1. Over a total of 2,107,300 sequences of different SARS-CoV-2 variants from GISAID, the *UtrechtU-ORF3a* forward primer has an accuracy of 98.15%, and the reverse primer of 97.99%. In Fig. 5 ( right) we can see the expected result that the created primer does appear in almost all sequences from B.1.1.7, B.1.1.529 and other lineages.

#### Laboratory testing

Next, in the **Hospital Civil de Guadalajara, México** we select 20 samples from patients, to perform the evaluation of the AI-designed RT-qPCR tests. The molecular diagnosis in this hospital was carried out with the *DeCoV19 Kit Triplex* (Genes2Life) endorsed by the Institute of Epidemiological Diagnosis and Reference (InDRE) in Mexico. We choose 15 positive and 5 negative nasopharyngeal and oropharyngeal swabs from patients with acute respiratory tract infection. The samples are tested with *DeCoV19 Kit Triplex*, *GeneFinder COVID-19 PLUS RealAmp Kit*, and the AI-designed primers *UtrechtU-ORF3a*.

Then, viral RNA is extracted from nasopharyngeal and oropharyngeal swabs using Qiamp Viral RNA mini kit. Approximately 75 µL of viral RNA is recovered and tested immediately. The DeCoV19 Kit Triplex detects 3 regions of the N gene and the human *RNAse P* gene. The GeneFinder COVID-19 PLUS RealAmp Kit detects regions of the *N*, *E*, and *RdRp* genes and an internal control. The AI designed primers bind to the *ORF3a* gene obtaining a 179 bp amplicon, this primers set are identified as *UtrechtU-ORF3a* primers. For commercial Kits, RT-qPCR is performed following the manufacturer’s recommendations, while for *UtrechtU-ORF3a* primers SuperScript III Platinum One-Step qRT-PCR Kit, forward (**5′-TAG CAC TCT CCA AGG GTG TTC-3′**), reverse (**3′-GCA AAG CCA AAG CCT CAT TA-5′**) and TaqMan probe (**5′-FAM CCT TGA AGC CCC TTT TCT CT NFQ-3′**) are used. All the RT-qPCR tests are developed in QuantStudio 5 Real-Time PCR System (Applied Biosystems).

Finally, to evaluate PCR efficiency we run a duplicate 10-fold dilution series of viral RNA for each assay using Twist Synthetic SARS-CoV-2 RNA Control 1. We calculate the slope by linear regression and defined the required levels for efficiency $>80\%$ and $R^2 \ge 0.98$, respectively. Thereafter, for the *UtrechtU-ORF3a* primers all the samples were evaluated with a replicate to obtain an indication of clinical sensitivity (n = 2). Furthermore, we calculated the viral load of samples according to standard curve of Twist Synthetic SARS-CoV-2 RNA Control 1.

### Experimental evaluation of B.1.1.7 Primer specificity

#### Create primer Set

**Figure 6:**
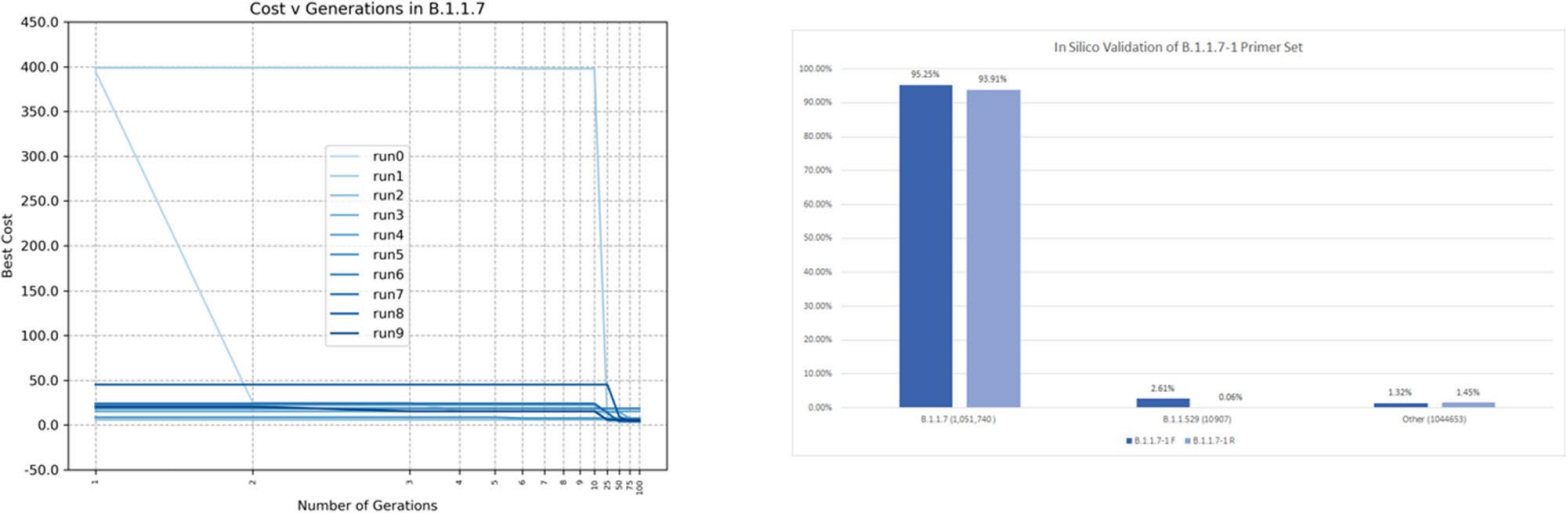
(Left) Cost function in 10 runs of the EA for 100 generations to find a forward primer in the the variant B.1.1.7. (Right) Percentage of appearance of the primer set in the 2,107,300 SARS-CoV-2 sequences.

We downloaded 10,712 SARS-CoV-2 sequences from the GISAID repository on December 23, 2020. After removing repeated sequences, we obtained a total of 2104 sequences labeled as B.1.1.7 and 6819 sequences from other variants, for a total of 8923 samples. Next, we select 1000 sequences for training the algorithm, where 605 are B.1.1.7 and 395 are other SARS-CoV-2 variants. Then, we assigned label 1 to B.1.1.7 variant samples, and the rest are assigned label 0, and we ran the EA algorithm 10 times, Fig. 6 (left). The results are reported in supplementary table 2.

Again, we simulated the candidate primers using Primer3Plus and EPI_ISL_601443 as the reference sequence for B.1.1.7. From the results, we selected the forward primer **5′-CAT GCT ATC TCT GGG ACC AAT-3′**, because the sequence has more than 1 single nucleotide mutation, and therefore has a higher probability of being a successful specific primer. In addition, given the position of the proposed reverse primer, we could use the Y144 deletion as reverse primer **3′-TGT TGT TTT TGT GGT AAA CAC C-5′** by displacing the result 10 bp. This results in an amplicon product size of 244 bp. This primer set is identified as *B.1.1.7-1*.

#### In silico validation

From an in-silico analysis, the *B.1.1.7-1* forward primer, tested on 1,051,740 B.1.1.7 sequences, has a sensitivity of 95.25% and specificity of 98.67%. The forward primer appears in 45% of Omicron BA.3, 61% of Omicron BA.5, and 69% of B.1.258 variant samples, that all did not exist when the primer was created. The reverse primer in the set does not appear in these lineages, although it appears in 22% of B.1.526 variant samples. Nevertheless, the forward and reverse primers appear at the same time only in the B.1.1.7 lineage, meaning that the primer set can be still effectively used to detect B.1.1.7, at least at the time of writing (June 2022). From Fig. 6 (right), we can see that forward and reverse sequences appear mostly in B.1.1.7 sequences, as expected, in comparison to samples from B.1.1.529 and other lineages.

#### Laboratory testing

Amplification efficiency of the designed primer sets was evaluated using viral RNA extracts from two sequenced SARS-CoV-2 strains: the original Wuhan strain 210207 (GISAID $\text {N}^{\circ }$ EPI_ISL_437689) and VOC B.1.1.7 strain (GISAID $\text {N}^{\circ }$ EPI_ISL_683466) in the **Pasteur Institute**. Viral RNA was extracted from infected cell culture supernatants using the NucleoSpin Dx Virus kit (Macherey-Nagel), following the manufacturers’ protocol. Viral RNA extracts (5 µL) were analyzed either using the IP2/IP4 dualplex real-time reverse-transcriptase (RT)-PCR assay, developed by following the Pasteur Institute and targeting conserved regions of the SARS-CoV-2 *RdRP* gene^31^, or primer set B.1.1.7-1, using the LightCycler EvoScript RNA SYBR Green I Master kit (Roche). Both RT-PCR assays were conducted on a LightCycler® 480 System (Roche), using the thermal cycling program described in the Pasteur Institute protocol^31^.

### Experimental evaluation of B.1.1.529 primer specificity

#### Create primers

**Figure 7:**
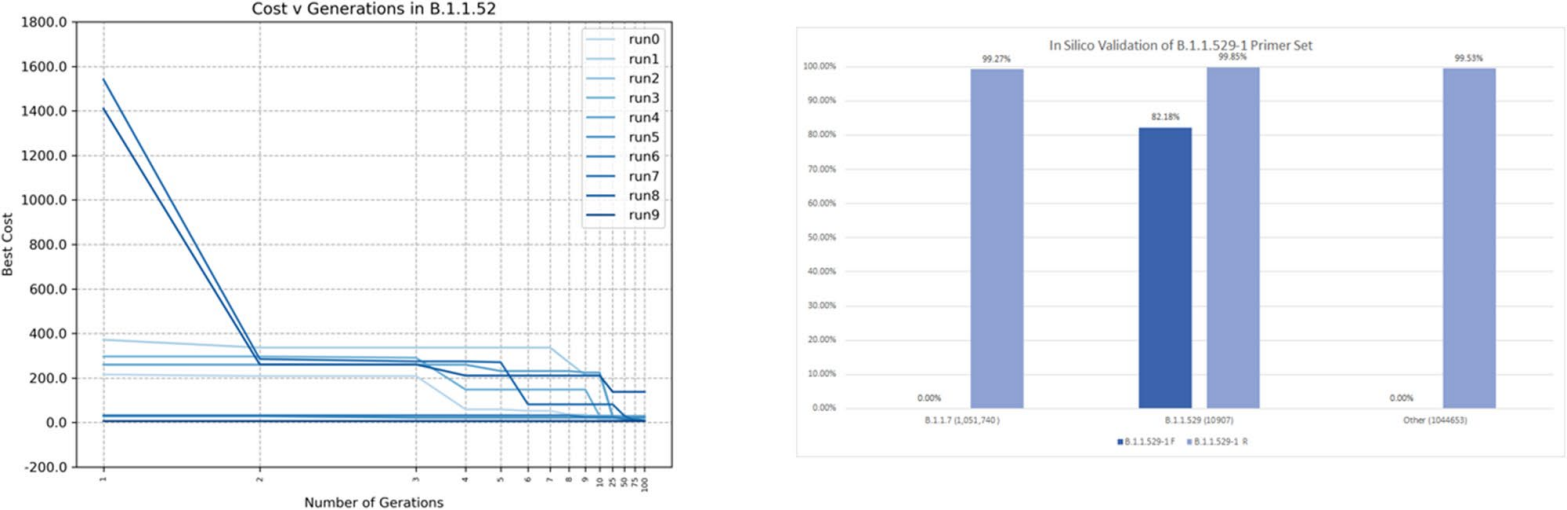
(Left) Cost function in 10 runs of the EA for 100 generations to find a forward primer in the the variant B.1.1.529. (Right) Percentage of appearance of the primer set in the 2,107,300 SARS-CoV-2 sequences.

From the GISAID repository^26^, we downloaded a total of 2100 sequences, in *.fasta format, with 123 sequences identified as B.1.1.529 (Omicron) and 100 sequences of each of the following variants, labeled following the Pango lineage^46^: AY.3 (Delta Sublineage), B.1, B.1.1.7 (Alpha), B.1.1.214, B.1.1.519, B.1.2, B.1.160, B.1.177, B.1.177.21, B.1.221, B.1.243, B.1.258, B.1.351 (Beta), B.1.427, B.1.429, B.1.526, B.1.596, B.1.617.2 (Delta), D.2, P.1 (Gamma), and R.1. Using EAs we found candidate forward primers from 10 runs of the algoritm, Fig. 7 (left). The results are reported in supplementary table 3.

#### In silico validation

Then, we simulated the candidate forward primers using Primer3Plus^28^ using the accession $EPI\_ISL\_6590782$ as reference for B.1.1.519 (Omicron) variant. Although sequence **GACCCACTTATGGTGTTGGTC** resulted in a warning for *High end self complementarity*, it presented 3 characteristics mutations of the B.1.1.529 (Omicron) variant^47^: Q498R (A23055G), N501Y (A23063T) and Y505H (T23075C), position 23,054 to 23,075 in the reference accession NC_045512.2^48^. A single-nucleotide mutation may not be enough to work as a specific primer for detecting SARS-CoV-2 variants. Thus, to solve the *High end self complementarity*, we increased the size of the primer by adding a base pair at the end (**GACCCACTTATGGTGTTGGTCA**), which resulted in an acceptable primer candidate with a $T_m$ of 62.0 $^{\circ }\text {C}$. We then generated the internal probe **CACCAGCAACTGTTTGTGGA** and reverse primer **CTGCCAAATTGTTGGAAAGG** with a $T_m$ of 60.8 $^{\circ }\text {C}$ and 60.5 $^{\circ }\text {C}$ respectively, with a product size of 208 bp. This primer set is identified as *B.1.1.529-1*.

The *B.1.1.529-1* forward primer, tested in-silico on 10,907 B.1.1.529 (Omicron) sequences, including the 5 sub-lineages, shows a sensitivity of 82.17%, and a specificity of 100% when tested on 2,096,393 sequences of other SARS-CoV-2 variants. The lower sensitivity is due to fact that several of the BA.1 samples in the repository contain sequencing errors (N) in the part of the genome that should match the forward primer. As seen on Fig. 7 (right) the reverse primer do appear in almost all SARS-CoV-2 sequences. Nevertheless, it is necessary to have both primers in order to give a positive.

#### Laboratory testing

To test the specificity of the primer set, raw saliva samples, approximately 1-2 ml, were collected in 5 ml screwcap containers from volunteers in the UniCoV study^32^ from the **University College Cork**. Following collections samples were heat inactivated at 95 °C for 5 min, cooled, vortexed and then 20 ml of saliva was added to 20 ml of Saliva Ready$^{\textrm{TM}}$ Solution in a 0.2 ml 96 well plate. The plate was vortexed and centrifuged and then heated at 62 °C for 5 min, 92 °C for 5 min and then cooled at $4^{\circ }\text {C}$. Saliva was then screened for SARS-CoV-2 (*ORF1a*, ORF1b and *N* gene) and Human *RNase P* using the TaqMan$^{\textrm{TM}}$ 1-Step Mutiplex SARS-CoV-2 Fast PCR Kit 2.0 on the Applied Biosystems$^{\textrm{TM}}$ QuantStudio$^{\textrm{TM}}$ 5 Real Time PCR Instrument, 96 well, 0.2-mL block (Thermo Fisher Scientific) according to the manufacturer’s instructions including a Positive Control: reverse transcription 53 °C for 5 min, 1 preincubation 85 °C for 5 min, activation at 95 °C 2 min with 40 cycles of denaturation 95 °C for 1 second and anneal/extension 62 °C for 30 seconds.

Positive SARS-CoV-2 samples were subsequently screened for the presence of the Omicron variant using our specific Omicron primer set (*B.1.1.529-1*). Briefly cDNA was synthesised using saliva from the Saliva Ready$^{\textrm{TM}}$ step above with LunaScript RT-Supermix (NEB), briefly for primer annealing for 2 min at 25 °C , cDNA synthesis for 10 minutes $55\,^{\circ }\text {C}$ 2 minutes and denaturation 95 °C for 1 minute. qPCR using followed by second and anneal/extension 62 °C for 30 seconds.

Finally, qPCR was performed using cDNA from above with the Luna Universal Probe qPCR mix (NEB), with Omicron specific primers and N1 (2019-nCoV RUO) primers/probes (Integrated DNA Technologies (IDT)) at a concentration 500 nM with probes at 250 nM(FAM-labelled). PCR conditions are denaturation at 95 °C fro 1 min and 40 cycles of denaturation 95 °C for 15 second and anneal/extension 60 °C for 30 seconds on the Applied Biosystems$^{\textrm{TM}}$ QuantStudio$^{\textrm{TM}}$ 5 Real Time PCR Instrument, 96 well, 0.2-mL block. All PCR reactions were performed in duplicate with a technical replicate performed following initial analysis.

### Supplementary Information

Supplementary Information 1.

## Data Availability

All generated primers, results, and code, are available at: https://github.com/steppenwolf0/EACovid.
