# Supplementary material for: An Innovative AI-based primer design tool for precise and accurate detection of SARS-CoV-2 variants of concern
Source: Sci Rep. 2023 Sep 22;13:15782. doi: 10.1038/s41598-023-42348-y (PMC10516913; doi:10.1038/s41598-023-42348-y)
Supplement: Supplementary file 1 — Supplementary Information 1. [file 41598_2023_42348_MOESM1_ESM.pdf]

Supplementary information

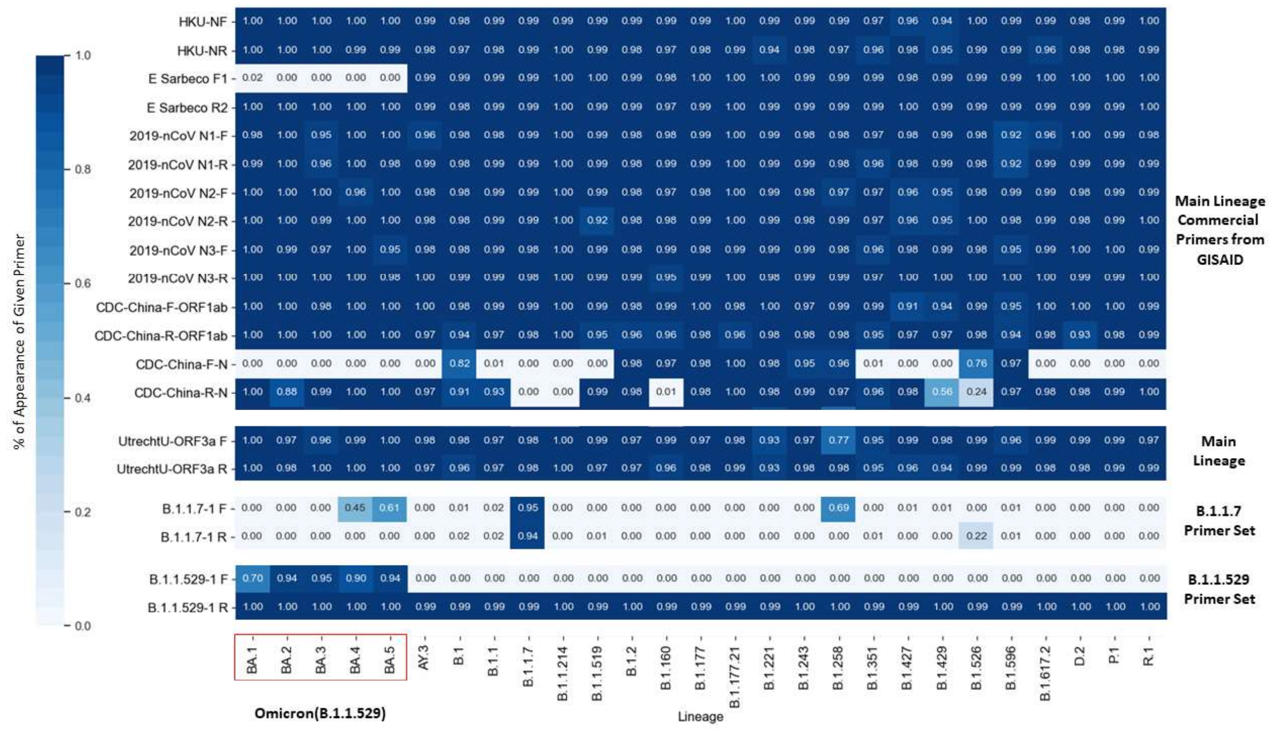

Supplementary Figure 1. Frequency of appearance of the different primer sets in 2,107,300 SARS-CoV-2 sequences downloaded from GISAID in June 11th, 2022. For the frequency we included primer sets suggested by GISAID. The "F" means forward sequences, whereas the "R" means reverse sequence.

Supplementary Table 1. Results of the 20 runs to find a SARS-CoV-2 primer set from the main lineage. When a candidate forward primer is successful, the Primer3Plus results will show a candidate reverse primer.

Candidate	Position in Refence NC_045512.2	Primer3Plus Results
GTTCGCGCTACTGCAACGATA	25477	Left primer is unacceptable: Tm too high
TTCGCGCTACTGCAACGATAC	25478	Left primer is unacceptable: Tm too high
CGCTACTGCAACGATACCGAT	25482	Left primer is unacceptable: High end self complementarity/High 3' stability
CGATACCGATACAAGCCTCAC	25493	AGAGAAAAGGGGCTTCAAGG
CGATACCGATACAAGCCTCAC	25493	AGAGAAAAGGGGCTTCAAGG
CGATACAAGCCTCACTCCCTT	25499	Left primer is unacceptable: High 3' stability
GGCGTTGCACTTCTTGCTGTT	25537	Left primer is unacceptable: Tm too high
AGATGGCAACTAGCACTCTCC	25594	GCAAAGCCAAAGCCTCATTA
CAACTAGCACTCTCCAAGGGT	25600	Left primer is unacceptable: High 3' stability
CAACTAGCACTCTCCAAGGGT	25600	Left primer is unacceptable: High 3' stability
AACTAGCACTCTCCAAGGGTG	25601	Left primer is unacceptable: High end self complementarity/High 3' stability
CTAGCACTCTCCAAGGGTGTT	25603	Left primer is unacceptable: High end self complementarity
**TAGCACTCTCCAAGGGTGTTC**	25604	**GCAAAGCCAAAGCCTCATTA**
**TAGCACTCTCCAAGGGTGTTC**	25604	**GCAAAGCCAAAGCCTCATTA**
**TAGCACTCTCCAAGGGTGTTC**	25604	**GCAAAGCCAAAGCCTCATTA**
**TAGCACTCTCCAAGGGTGTTC**	25604	**GCAAAGCCAAAGCCTCATTA**
CACACCTTTTGCTCGTTGCTG	25667	Left primer is unacceptable: Tm too high
CACACCTTTTGCTCGTTGCTG	25667	Left primer is unacceptable: Tm too high
ACACCTTTTGCTCGTTGCTGC	25668	Left primer is unacceptable: Tm too high/High 3' stability
ACCTTTTGCTCGTTGCTGCTG	25670	Left primer is unacceptable: Tm too high

Supplementary Table 2. Results of the 10 runs to find a B.1.1.7 primer. When a candidate forward primer is successful, the Primer3Plus results w will show a candidate reverse primer.

Candidate	Target Mutation	Primer3Plus Results
CATGCACTTTGTCTGAACAAC	C913T	Left primer is unacceptable: Tm too low
**CATGCTATCTCTGGGACCAAT**	HV 69-70 deletion (ACATGT21766-21771del)	**TGGTAAACACCCAAAATGGA**
GGCAGAGACATTGATGACACT	A570D (C23271A)	Left primer is unacceptable: High end self complementarity
GGCAGAGACATTGATGACACT	A570D (C23271A)	Left primer is unacceptable: High end self complementarity
ACTACACACAACACATTTGTG	D1118H (G24914C)	Left primer is unacceptable: Tm too low/High end self complementarity
CAACTCCAGGCAGCAGTAAAC	R204,G205R (G2881A,G2882A,G2883C)	GCCTCAGCAGCAGATTTCTT
CCAGGCAGCAGTAAACGAACT	R204,G205R (G2881A,G2882A,G2883C)	GAAGCCTCAGCAGCAGATTT
GCAGCAGTAAACGAACTTCTC	R204,G205R (G2881A,G2882A,G2883C)	Left primer is unacceptable: Tm too low
CCAGCTTGAGAGCAAAATGTT	S235F (C28977T)	Left primer is unacceptable: High end self complementarity
TGTTTGGTAAAGGCCAACAAC	S235F (C28977T)	CCCAAAATTTCCTTGGGTTT

Supplementary Table 3. Results of the 10 runs to find a B.1.1.529 primer. When a forward primer is successful, the Primer3Plus results will show a candidate reverse primer.

Candidate	Target Mutation	Primer3Plus Simulation
ATCTCTATCACCTCAGCTGTT	T492I (C10029T)	Left primer is unacceptable: Tm too low/High end self complementarity
TACCAATGTGCTATGAGGCAC	P132H (C10449A)	Left primer is unacceptable: High end self complementarity/High 3' stability
TGGCCAGAGGTGTTGTTTTTA	I189V (A11537G)	GGTGGGAGTAGTCCCTGTGA
CTGGTCAGGCAATAACAGTCA	T13195C	ACCCACAGGGTCATTAGCAC
AGGCAATAACAGTCACACCGG	T13195C	Left primer is unacceptable: High end self complementarity/High 3' stability
GTCACACCGGAAGCCAATATG	T13195C	ACCCACAGGGTCATTAGCAC
**GACCCACTTATGGTGTTGGTC**	Q498R (A23055G), N501Y (A23063T) and Y505H (T23075C)	Left primer is unacceptable: High end self complementarity
TAGATTCATTCAAGGAGGAGT	C25000T	Left primer is unacceptable: Tm too low
AGAGATAGGTACGTTAATAGT	T9I (C26270T)	Left primer is unacceptable: Tm too low
ACTTGTTTTGTGCTTGCTGCT	A63T(G26709A)	TTGAATGACCACATGGAACG

Supplementary Table 4. Number of sequences used for the *in-silico* analysis from the GISAID repository.

Lineage	Sequences	Lineage	Sequences
BA.1	5,445	B.1.177	74,475
BA.2	4,908	B.1.177.21	13,049
BA.3	92	B.1.221	13,510
BA.4	77	B.1.243	12,841
BA.5	385	B.1.258	14,061
AY.3	17,504	B.1.351	30,650
B.1	82,955	B.1.427	18,239
B.1.1	47,373	B.1.429	39,345
B.1.1.7	1,051,740	B.1.526	49,786
B.1.1.214	18,066	B.1.596	10,939
B.1.1.519	23,376	B.1.617.2	366,831
B.1.2	101,442	D.2	12,772
B.1.2	101,442	P.1	59,692
B.1.160	27,353	R.1	10,394
		Total	2,107,300

Supplementary Table 5. Results of the 20 runs to find a human Monkey Pox primer set. When a forward primer is successful, the Primer3Plus results will show a candidate reverse primer.

**Candidate**	**Position in reference EPI_ISL_13053218**	**Primer3Plus Simulation**
CCATGTCTGAAACGAGACGCT	26,279	Left primer is unacceptable: High 3’ stability
CTGATAGAATGCGAGCAGCGA	38,536	Left primer is unacceptable: Tm too high/High 3’ stability
TACAGTGATGCCTACATGCCG	43,945	Left primer is unacceptable: High 3’ stability
GCATGGTACGTCTGACAACAC	58,339	TCACCATGCAATCTGGTGAT
AAGAAGCGAACGCTAGTGCTC	77,261	Left primer is unacceptable: High end self complementarity
TGTGTGGATTTCGGCGCCTAT	88,203	Left primer is unacceptable: Tm too high/High end self complementarity
GTGTTCTGCAACAAGACGAGG	88,707	Left primer is unacceptable: High 3’ stability
GTGTTCTGCAACAAGACGAGG	88,707	Left primer is unacceptable: High 3’ stability
TCCCCGGTAACTACCATAGTG	98,271	ACCTCTAATAGCCGCATCCA
ATATCATCCAGCGGCCAGAGA	98,294	Left primer is unacceptable: Tm too high
GATGTATGGTGCAGCCTGAAG	112,242	AACGATGATGATGCGGTAGA
GTGTCTCCATGTACATACGCG	112,636	Left primer is unacceptable: High end self complementarity/High 3’ stability
CGTTCGTCACCAGATCGTTTG	113,538	Left primer is unacceptable: Tm too high
AAACGGAACTTCCGTACGACG	134,017	Left primer is unacceptable: Tm too high/High self complementarity/High end self complementarity/High 3’ stability
CCTCCAGTAGCATGTGGTTCT	136,960	Left primer is unacceptable: High end self complementarity
CCTCCAGTAGCATGTGGTTCT	136,960	Left primer is unacceptable: High end self complementarity
TCCGCTACTGTTTACGGAGAC	143,087	Left primer is unacceptable: High end self complementarity
CCAACACGTGCATCCATGCAA	150,692	Left primer is unacceptable: Tm too high/High end self complementarity
AACGAGGGTTCCATGGCAGAT	155,882	Left primer is unacceptable: Tm too high
ACGACCTAGACGCCCTACAAA	183,169	TGGTCTAGGGGGAAGTTGTG
